# The quantification of bisphenols and their analogues in wastewaters and surface water by an improved solid-phase extraction gas chromatography/mass spectrometry method

**DOI:** 10.1007/s11356-020-09123-2

**Published:** 2020-05-16

**Authors:** Magda Caban, Piotr Stepnowski

**Affiliations:** Department of Environmental Analysis, Faculty of Chemistry, University of Gdansk, ul. Wita Stwosza 63, 80-308 Gdańsk, Poland

**Keywords:** Bisphenol A, Bisphenol analogues, Multi-component analysis, Wastewater monitoring, Silylation

## Abstract

The study focused on the application of GC in the quantitative analysis of bisphenols and their analogues (12 analytes), and the improvement of solid-phase extraction for the whole water analysis of complex water samples. The role of silylation in the qualitative and quantitative analysis of bisphenols was investigated. Partial degradation occurred for selected targets during hot injection with the presence of a silylation agent. A PSA (primary and secondary amines) sorbent placed on the top of the solid-phase extraction (SPE) column sorbent was found to be a matrix component trap, mostly for humic acids. The whole water analysis was performed by washing the filters with methanol and recycling the extract to the sample. The validation of SPE-GC/MS(SIM) gave limits of detection of 1–50 ng/L for ten target bisphenols with a method recovery of between 87 and 133%. The application of the method was tested by the analysis of wastewater sampled from three wastewater treatment plants located in Poland, and municipal surface waters. The only analytes found were BPA and BPS, within the range of 16–1465 ng/L and < MDL-1249 ng/L in wastewater, and 170–3113 ng/L and < MDL-1584 ng/L in surface water, respectively.

**Electronic supplementary material:**

The online version of this article (10.1007/s11356-020-09123-2) contains supplementary material, which is available to authorized users.

## Introduction

The increase in anthropogenic pressure on the environment with regard to hazardous substances is strictly connected with industrial development. However, the introduction of new materials, especially synthetic polymers, gives humanity new possibilities for development. The main applications of synthetic polymers are food packaging, the building industry, the electrical and electronics industry, daily life items (furniture, toys, fibers, clothes), and many more. One of the compound which can be found in plastics is bisphenol A (BPA), a chemical with endocrine-disrupting potential (Corrales et al. 2015; Vahedi et al. 2016; Murphy et al. 2019). BPA, discovered in 1891, is a monomer for the synthesis of selected polycarbonates (PC), epoxy and vinyl ester resins, and polysulfones. Most of the items containing BPA are made from rigid, transparent and temperature-/pressure-stable polymers. Epoxy resins are used mostly as protective layers, for example, inside cans and water pipes, or in paper used in sales receipts. In 2015, BPA production was 4 million tonnes, which was the largest amount for a single compound (Almeida et al. 2018). Moreover, derivatives and analogues of BPA are also produced. Currently, BPA is a compound of high interest because it is classified as an endocrine-disrupting chemical (EDC) and is proven to leach from packaging materials into food. “BPA-free” items are marketed as safe. Nevertheless, although the substitution of BPA by BPA-like compounds can be achieved (Chen et al. 2016), statistics of its production and use are hard to find. What can be found in the literature is that often, instead of BPA, such analogues are used as bisphenol S (BPS), bisphenol F (BPF), and bisphenol AF (BPAF) (Warner and Flaws 2018). In the thermal paper, 19 bisphenols (BPs) were identified (U.S. Environmental Protection Agency 2015). Scientific reports show the similar endocrine-disrupting potential of other bisphenols (Warner and Flaws 2018; Wang et al. 2019) and the ecotoxicological risk of mixtures of bisphenols (BPs) (Owczarek et al. 2018). BPF was found to exhibit genotoxicity (Cabaton et al. 2009). All these facts raise concern regarding the safe use of alternatives to bisphenols. In addition to human health safety, the environmental impact is also observed (Corrales et al. 2015). The main route of BPs into the environment is wastewater (WW), both residential and industrial. The detection frequency and concentration level are generally known for BPA (up to 84,000 ng/L in raw WW) (Gercken et al. 2018; Wang et al. 2019), while for BPA-like compounds, only a few reports have been presented, which found concentrations were much lower (dozens to hundreds ng/L) (Sun et al. 2017; Česen et al. 2018; Wang et al. 2019; Xue and Kannan 2019). This limitation can be caused by problems in application of one technique for the problematic analysis of several BPs in one run. LC-MS and LC-MS/MS are often used for such analyses, but some troubleshooting can occur (for example, the presence of BPA in the solvents and the system, the instability of some analogues in solutions) (Wilczewska et al. 2016; Szczepańska et al. 2019). GC-MS was rarely used for the analysis of BPA and its analogues because of the need for silylation. Nevertheless, this process of derivatization could be beneficial for the quantification of organic pollutant traces in natural waters (Caban et al. 2011; Sadkowska et al. 2017). To date, the advantages and limitations of the application of GC/MS for the quantification of BPs have not been investigated.

The aim of this work is to verify the use of the GC-MS technique in order to perform the quantification of twelve BPs (targets presented in Table S1, Supplementary Information). The next objective was to test a modification of solid-phase extraction (SPE) for whole water analysis in samples with a high content of dissolved organic matter, in which BPs can be adsorbed (in most available reports, only the free fraction was determined). Thanks to this, it was aimed to examine the current level of BPs in an exemplary urban area samples in which such a study has never been performed before, but it is known to be strongly impacted by human activities. In addition, the WW samples were taken from three wastewater treatment plants (WWTP), differentiated in size and technologies, in order to check the presence of BPs other than BPA (rarely investigated).

## Experimental

### Chemicals and materials

Standards of BPs (BPE, bisphenol E; BPC, bisphenol C; BPA-DMC, bisphenol A dimethacrylate; BPBP, bisphenol BP; BPF, bisphenol F; BPA-DGE, bisphenol A diglycidyl ether; BPA-DAC, bisphenol A diacetate; BPZ, bisphenol Z; BPFL, bisphenol FL; BPAF, bisphenol AF; BPS, bisphenol S; BPA, bisphenol A; BPA-D16, deuterated bisphenol A) were purchased from Sigma-Aldrich, with a minimum of 98% purity. Stock solutions were prepared in methanol (HPLC purity, POCH, Poland) and stored at – 20 °C. Working solutions were also prepared in methanol. During the experiments, all equipment used was made of glass or Teflon (except the SPE cartridge, made from polypropylene, PP). Derivatization was performed using a BSTFA (*N*,*O*-bis(trimethylsilyl)trifluoroacetamide) + 1%TMCS (trimethylchlorosilane) reagent (*Synthese North*, Germany).

### Pretreatment and solid-phase extraction of water samples

Several sets of solid-phase extraction (SPE) were tested (Table 1). The differentiating factors were (a) the addition of humic acids (HA, 5 mg/L) as representatives of dissolved organic matter, (b) the filtration of the sample before SPE using glass fiber filters, (c) the washing of the filters with 2 × 5 mL of methanol and adding the obtained solution to the water sample, (d) the addition of 50 mg of the PSA (primary and secondary amines, Supelco) sorbent on the top of the Strata-X column (200 mg/3 mL, Phenomenex) to retain some of the water sample matrix. The internal standard (IS) was added to the water samples before any treatment of the water sample. For the determination of the extraction efficiency, the mixture of analytes was added to obtain a concentration of 4 μg/L in the samples. The SPE protocol was as follows: pre-washing of the column with 3 mL of methanol, followed by the application of 3 mL of deionized water, the application of 100 mL of the sample using vacuum assistance, washing with 3 mL of 5 % aq. methanol, drying, washing with 3 mL of hexane (HPLC grade), drying, and elution with 3 + 3 mL of methanol. The extract was concentrated and transferred to chromatographic vials and the solvent was totally removed. The dry extracts were stored at – 20 °C.

**Table 1:** The sets tested under the optimization of solid-phase extraction (SPE) (HA—5 mg/L of humic acid in the water sample), PSA - sorbent based on primary and secondary amines)

Sets	Filtration before SPE	Washing of the filters with MeOH and the addition of methanolic extract to the water sample	The addition of PSA on top of the SPE column
1	Yes	No	No
1HA			
2	Yes	Yes	No
2HA			
3	Yes	Yes	Yes
3HA			
4	Yes	No	Yes
4HA			
5	No	No	No
5HA			

### Derivatization and GC/MS(SIM) analysis

For derivatization, the solvent was removed from the extract, 50 μL of the reagent was introduced and the vials were vortex mixed. The reaction of trimethylsilyl (TMS) derivatives synthesis was at 30 °C, while the time was 30 min. The optimization of these parameters was performed for BPs, and the results show that prolonging the reaction time to 60 min or increasing the temperature to 75 °C and 90 °C did not change the reaction success (the surface area of the chromatographic peak was stable, Fig. S1 in Supplementary Information). After derivatization, the post-reaction solution was transferred to glass inserts closed by caps with red Teflon/silicone membranes, and the samples were subjected to analysis using the GC/MS equipment (GC-2010 Plus coupled to a GCMS-QP 2010 SE mass spectrometer (Shimadzu)). The GC parameters were as follows: column 30 m × 0.25 mm × 0.25 μm (ZB-5, Zebron), injection 305 °C, constant helium pressure 100 kPa, injection volume 1 μL, splitless per 1 min, temperature program: starting temperature 120 °C per 1 min, then a rate of 10 °C/min, final temperature 300 °C per 6 min (total 25 min), autosampler washing solvent—acetone. The transfer line was heated to 305 °C. The MS analysis was performed using the following settings: EI (70 eV) temperature 200 °C, solvent cut time 5 min, scan 45–800 *m/z*, or SIM (selected ion monitoring), 3 scans per second. Instrument calibration and tuning were performed before each sequence run (normally—daily). The qualitative analysis was performed using (a) the characteristic retention time (span of ± 0.2 min); (b) a min of two *m/z* values, from which one was the quantitative ion and the rest were the confirmation / reference ions; and additionally, (c) the ratio of quantitative ions to confirmation ions (specified for the standards). The quantitative analysis was performed using the internal standard (IS, BPA-D16), introduced to each sample, in the same amount, and plotting the relative response of the analyte to the IS. Calibration was performed by an analysis of the pure standards (min. six points in the calibration curve, in duplicate) to check the linearity of the MS response in the tested concentration range, and the instrumental detection and quantification limits.

### The validation of the SPE-GC-MS(SIM) method

For the validation of the method, the latest version of the IUPAC (International Union of Pure and Applied Chemistry) guidelines for single-laboratory validation of methods of analysis (IUPAC 2002) suited the purpose of this study, with the support of general EURACHEM guidelines (CITAC/EURACHEM 2002). The validation was performed using the matrix-matched method by spiking the deionized water samples (test portion 100 mL, HA concentration of 5 mg/L) with the BPs to obtain the following concentrations: 1000 ng/L, 500 ng/L, 100 ng/L, 50 ng/L, 10 ng/L, 5 ng/L, 1 ng/L, 0.5 ng/L, 0.1 ng/L in duplicate during the same day. The IS was added to each of the samples with the same concentration. Then, SPE was performed according to the 3HA protocol (Table 1). The obtained calibration curves were used for the quantification of BPs in WW and SW samples.

Quality assurance (QA) was performed using a suitable laboratory environment, skilled staff, the calibration and tuning of equipment, and generally good laboratory practice (following EURACHEM (CITAC/EURACHEM 2002)). Quality control (QC) was performed by the analysis of blanks, spiked samples, and duplicates. In order to verify the SPE-GC-MS(SIM) method, the following parameters/characteristics were determined—applicability, selectivity, trueness, and precision. The applicability of the new method was the screening of a whole-water analysis of concentrations of mixtures of BPs (MDL 0.3–1.7 ng/L) in natural waters and wastewater. The selectivity description is as follows: the organic compounds dissolved in water have no impact on quantification; in the blank sample, BPA can be found, with a negligible impact on quantification. Accuracy was determined by trueness and the calculation of precision. Trueness was proved by the spiking/recovery method (no reference material available). Precision was calculated as the relative standard deviation (RSD, %) of concentrations found after the repetition of spiking. The matrix variation was tested by the spiking/recovery of deionized water, and raw and treated WW. The method specificity is the analysis of bisphenol-type compounds in the presence of other substances from this group.

For the method quantification limit (MQL), the following parameters were used: the lowest concentration with precision lower than 5% and method recovery between 70 and 130%, while for the method detection limit (MDL): MDL = MQL/3 + signal to noise ratio 3:1. Before each sequence of analysis, a blank (deionized water without the addition of BPs) was analyzed to track the residual of the concentration of BPs in solvents and the reagent use.

In addition to the validation of the SPE-GC/MS(SIM) method, the validation of only the instrumental analysis (without the extraction step) was performed to present the GC/MS possibilities in order of each of the twelve target BPs.

### The screening of bisphenols in surface water and wastewaters

The validated method was tested by the determination of BPs in raw and treated WWs sampled from three WWTPs in north Poland and from surface water (SW) in Gdansk streams and reservoirs. Gdańsk-Wschód WWTP is the biggest in the region with mechanical, biological, and chemical treatment technology (capacity 120,000 m^3^/day). The WWTP located in Gniewino is small but also with the same technology (capacity 850 m^3^/day). The WWTP in Swarzewo is a small plant with an SBR (sequencing batch reactor, capacity 7000 m^3^/day). The 24-h accumulated samples were collected in PE containers in December 2019.

The four grab samples of SW (100 mL of each) were collected in the city of Gdansk (the map of sampling points is presented in Fig. S2, Supplementary Information) in January 2020. Sample 1 was taken from Oruński Stream, sample 2 from Kozacki Stream, and both streams flow into the Świętokrzyska I retention tank, the outflow from which sample 3 was taken. The water flows from the Świętokrzyska I tank to the Świętokrzyska II retention tank, and the water was also taken from the outflow of this reservoir (sample 4). The water from this reservoir ends in a bigger river and finally in the Baltic Sea. The mentioned streams and reservoirs are located inside the city, and their main role is to collect storm water, thereby the presence of BPs is suspected. Furthermore, the Kozacki Stream is partially located under a municipal landfill.

## Results and discussion

### Qualitative and quantitative analysis of BPs by GC-MS

Each of the tested analytes and the internal standard (IS) were analyzed with and without derivatization. Three bisphenol A-based compounds—BPA-DMC, BPA-DGE, and BPA-DAC—cannot be derivatized by BSTFA, as they do not possess active hydrogen for replacement via silylation. For them, the chromatographic signal areas were similar when they were dissolved in toluene (without derivatization) or the BSTFA reagent. The rest of the analytes did not give signals in samples not subjected to derivatization. In the literature, a method of BPA analysis by GC without derivatization can be found (del Olmo et al. 1997; D.A. Markham et al. 1998; Oca et al. 2013), but using a cool injection technique, such as a programmed-temperature vaporizer or a cool on-column injector. In a modern split-splitless (S/SL) injection of a natural sample extract, it would be problematic to inject into a cool injector. Furthermore, the limits of detection of TMS derivatives are mostly lower than those for underivatized analytes (Caban et al. 2013b). For example, the detection limit obtained in our study was ng/L, while being 1000 times higher (μg/L) for a cool on-column injection of underivatized samples (D.A. Markham et al. 1998) and for the injection of underivatized samples into an S/SL injector heated to 200 °C (del Olmo et al. 1997). The analysis of BPA as a derivative of tert-butyldimethylsilyl (Durán-Alvarez et al. 2009) gives similar limits of detection as in our study.

In the case of BPA-DMC, it was observed that it is possible that methacrylate groups can be detached from native compounds, and the mono-TMS derivative of BPA-mono-methacrylate was observed in the chromatogram. The *m/z* value of the molecular mass of such a new compound was 368 *m/z*, with M-15 as the highest signal on the mass spectra (full spectrum presented in Fig. S3, Supplementary Information). The other signals were 69 and 73 *m/z*, proving that such a molecule has both a methacrylate and a trimethylsilyl group. The relative intensity of the peak to BPA-DMC was between 2.8 and 23.0% in three independent samples. In the chromatogram of the sample not subjected to derivatization, the only peak found was underivatized BPA-DMC. The presence of an additional peak suggests most probably that during derivatization or injection into a hot injector, BPA-DMC was partially decomposed. This reflects the quantification of BPA-DMC—a high quantification limit compared with most BPs and a lower regression coefficient of the curve (Table S2, Supplementary Information). Similarly, BPA-DAC was found to degrade partially with the release of the acetate group, and the obtained product was derivatized into BPA-mono-acetate-mono-*O*-TMS (BPA-AC-TMS, mass spectrum on Fig. S3, Supplementary Information). It is not clear where the degradation occurs—during derivatization or injection into the hot injector. BPA-DGE was also found to have an additional peak coming from the release of the one glycidyl-ether group, so-called BPA-GE. The mass spectrum of the BPA-GE-TMS derivative is presented in Fig. S3 (Supplementary Information). In the case of BPs with two hydroxyl groups subjected to derivatization, no additional chromatographic signals were obtained.

Table S2 (Supplementary Information) presents the selected parameters of the instrumental calibration curve using BPA-D16 as the internal standard (IS). Similar good calibration line regression coefficients, limits of detection, and precision were found for the BPs, despite derivatization being impossible for BPA-DMC, BPA-DGE, and BPA-DAC.

**Fig. 1:**
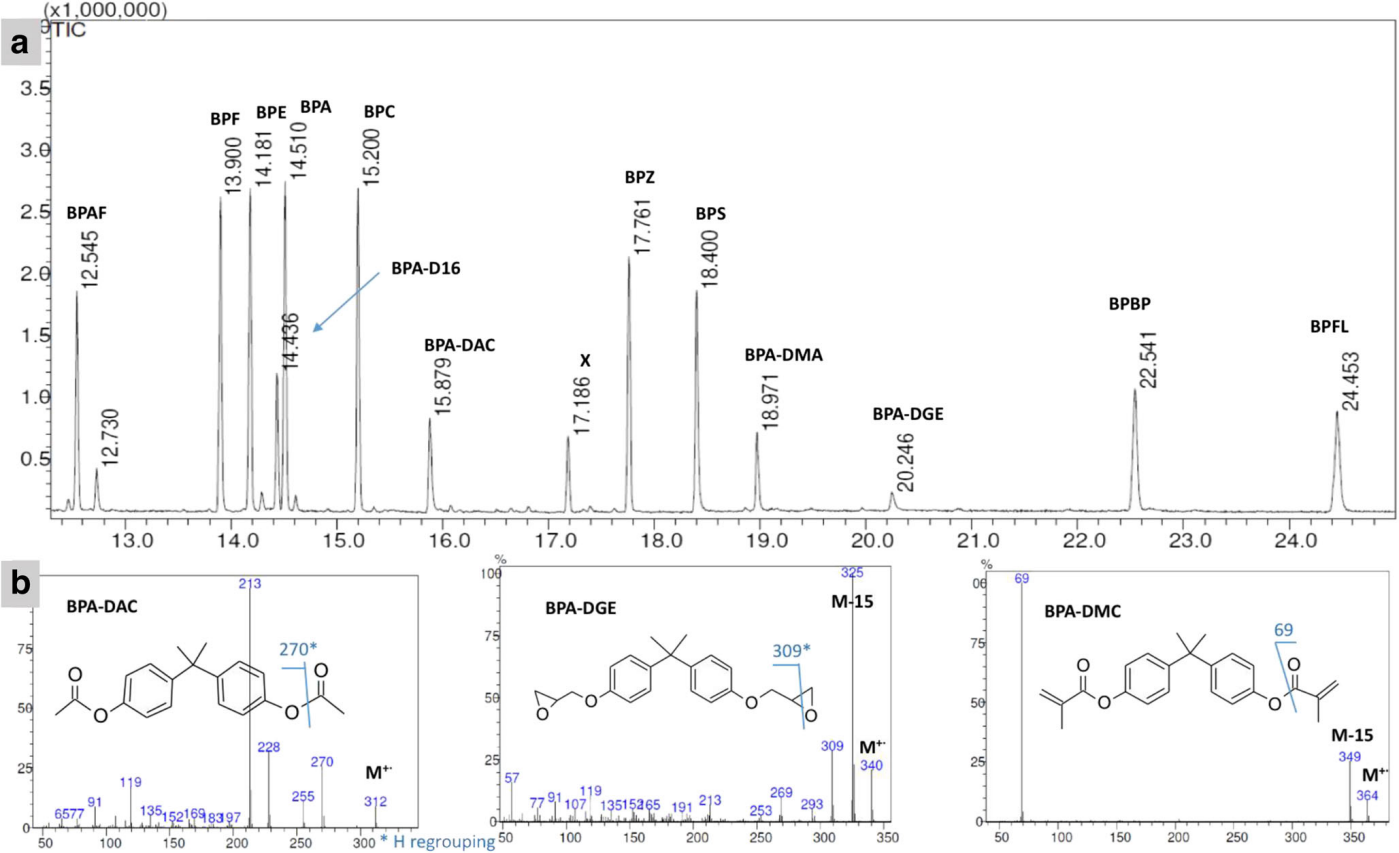
Part **a** The GC/MS chromatogram of the analysis of twelve bisphenol analogues together with deuterated bisphenol A. part **b** The mass spectra of bisphenols which could not be derivatized

Figure 1 presents the chromatogram of total ion current (TIC) of the GC-MS analysis of the solution of twelve analytes and the internal standard (2 μg/mL, for IS 1 μg/mL). The retention times of the BPs with only oxygen as a heteroatom depend on the *m/z* values of the molecular ions. The retentions of BPAF and BPS are out of this dependency. BPAF possesses fluorine atoms inside its structure and has the lowest retention times. The lowering of retention times due to the introduction of fluorine atoms was presented by us previously for pharmaceuticals (Caban et al. 2013a, 2013b, 2014). The longer retention time of BPS with respect to BPA as TMS derivatives in the capillary column was mentioned by Cao (Xu-Liang 2019), as a response to the incorrectly presented results of a previous work by Viñas et al. (2010).

**Fig. 2:**
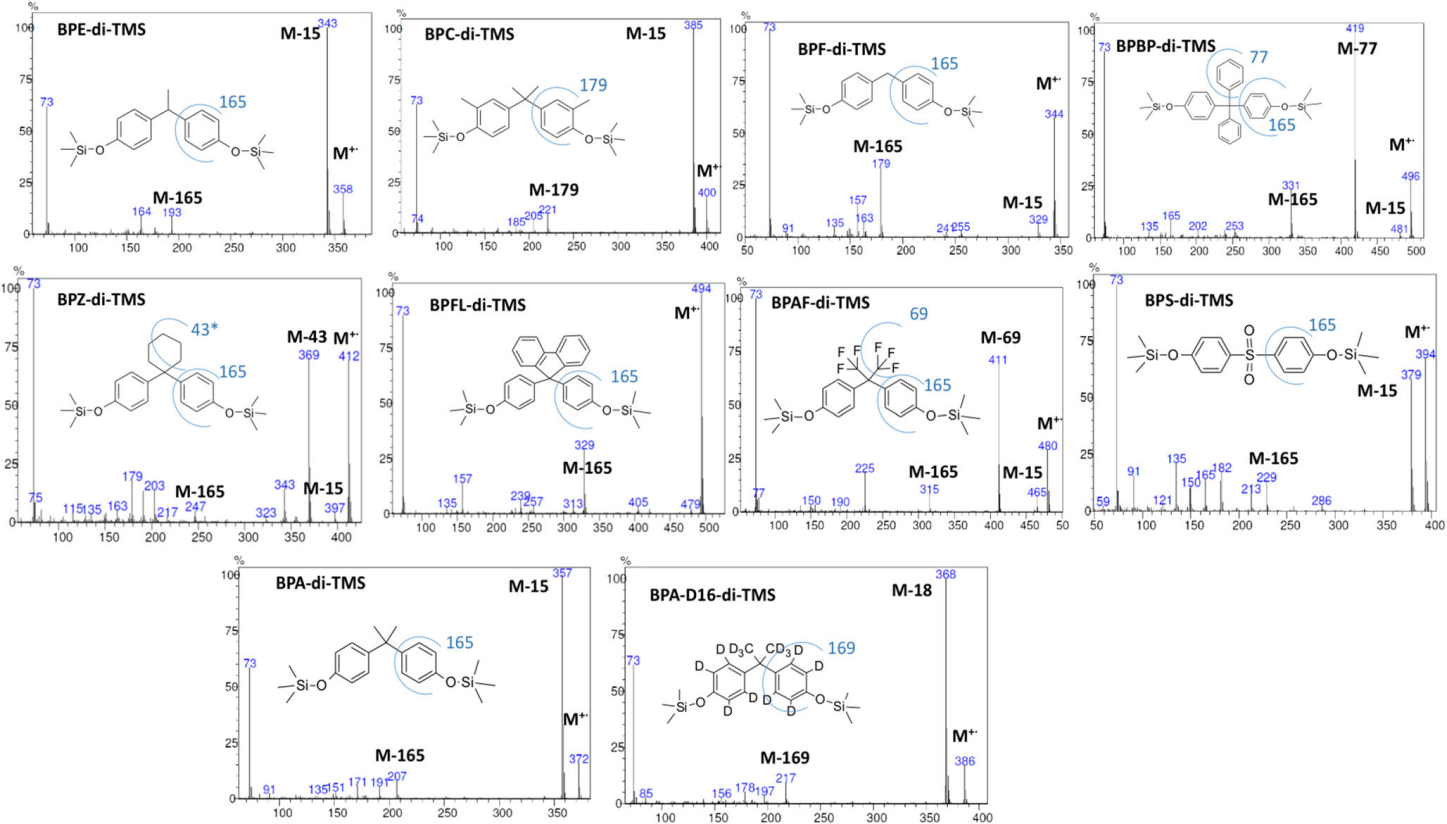
The mass spectra and main fragmentation pathway found of nine bisphenol analogues and deuterated bisphenol in the form of trimethylsilyl (TMS) derivatives

The TIC response of the detector (the general potential of analyte ionization) was comparable for BPAF, BPF, BPE, BPA, BPC, BPZ, BPS, BPBP, and BPFL as TMS derivatives (Fig. 2 presents the mass spectra of the TMS derivatives). The response of the detector for BPA-DAC and BPA-DMC was lower compared with the TMS derivatives, which can be caused by the lower opportunity for ionization. The better chromatographic response of silyl derivatives compared with underivatized compounds was presented by several authors, including ourselves (Caban et al. 2013a, 2013b, 2014). For underivatized BPA-DGE, a relatively polar compound, the detector response was much lower, which suggests decomposition or sorption inside the injector/column. Furthermore, previous studies show the instability of such a compound in methanolic solvent (Szczepańska et al. 2019). The lower TIC detector response also reflects a lower response in the selected ion monitoring (SIM) mode, and higher limits of detection for BPA-DMC, BPA-DGE, and BPA-DAC (Table S2 in Supplementary Information). The fragmentation pattern of the molecular ions was described in Section S1 in Supplementary Information.

Quantification was performed using the selected ion monitoring (SIM) mode. For the purpose of quantification, one of the signals in the spectrum was chosen, named as the quantitative ion. Qualification was performed using the retention times and reference ions (one, two, or three, depending on the compound). The additional confirming factor was the ratio of the quantitative/reference ions. The mentioned ions are presented in Table 2. Despite high intensity, *m/z* 73 was excluded from the SIM mode, as it is not characteristic.

**Table 2:** The validation parameters of the SPE-GC/MS(SIM) method for the analysis of ten bisphenols in water samples (sample volume 100 mL, internal standard—bisphenol A-D16)

Analyte qualitative/reference *m/z*	Correlation coefficient (MQL-1000 ng/L) (*R*^2^)	Method trueness–method recovery (*C*_found/_*C*_spiked_ × 100%) [%] ±RSD	Method precision (RSD) [%]	Method quantification limit (MQL) [ng/L]	Method detection limit (MDL) [ng/L]
BPAF 411/480, 465	0.9921	80–109 ± 7	1.0–7.3	1.0	0.3
BPF 344/157	0.9948	78–105 ± 11	0.8–7.4	5.0	1.7
BPE 343/358	0.9970	90–133 ± 9	0.1–6.9	1.0	0.3
BPA 357/372	0.9985	96–120 ± 8	1.0–6.0	5.0	1.7
BPC 385/400	0.9937	104–122 ± 2	0.6–9.3	5.0	1.7
BPA-DAC 213/228, 270, 312	0.9984	87–135 ± 13	3.0–13.0	50.0	17.0
BPZ 412/369, 343	0.9959	97–111 ± 7	0.6–7.7	1.0	0.3
BPS 394/379, 182	0.9990	87–104 ± 7	0.9–10.9	5.0	1.7
BPBP 419/331, 496	0.9909	90–111 ± 6	1.8–8.9	5.0	1.7
BPFL 494/329	0.9986	108–115 ± 3	2.6–10.1	5.0	1.7

Finally, it can be stated that from the twelve BPs, nine can be successfully analyzed by GC-MS, while three (BPA-DMC, BPA-DGE, BPA-DAC) should rather be analyzed by LC/MS. Nevertheless, it was decided to analyze BPA-DAC in WW samples along with the other nine easily silylated BPs.

### Solid-phase extraction performance

**Fig. 3:**
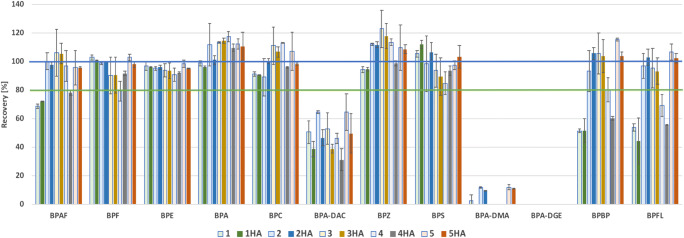
The extraction recovery [%] of the tested twelve bisphenols in relation to solid-phase extraction performance by ten sets (sets 1 to 5HA, presented in Table 1, the level of acceptable recovery of 80% and desired recovery of 100% were marked by lines)

The sorbent inside the Strata-X column is a copolymer of divinylbenzene and vinylpyrrolidone, thereby its adsorption properties are universal, and both polar and non-polar compounds should be retained. In our study, we tested two ways of sample pretreatment—samples were either filtered or not with glass fiber filters; the filters were washed or not with MeOH. The other modification was the application of the PSA sorbent at the top of the Strata-X column. The sample pH was not changed, as BPs are weak acids, and are non-ionized in natural waters. Figure 3 presents the values of extraction recovery (%) of the 12 tested BPs (relative to the IS). The descriptions of test sets (1–5) are presented in Table 1. The notation HA means that humic acids were added to the sample. The 3HA set corresponds to the whole water analysis of BPs in environmental water due to the following reason: the water is rich in dissolved organic matter with adsorption potential (Murray and Örmec 2018) (the similarity of the structure of HAs to BPs was another reason). The precipitated HAs were removed before SPE by the filtration of the sample with glass fiber filters to prevent the SPE column from being blocked (often observed for non-filtered WW samples), the adsorbed fractions of BPs were eluted from the filters to the samples with 5 mL of MeOH (the concentration of organic solvent inside the sample was not higher than 5%), then the full procedure of SPE was performed. Set 5 is the extraction of BPs from clear water without any pretreatment.

Generally, for BPF, BPE, BPA, BPC, BPZ, and BPS, the change in pretreatment and the addition of HAs have no effect on the extraction, and the extraction recovery was higher than 80%, defined as the acceptable level. For BPAF, BPBP, and BPFL, it was noted that the addition of washing the filter is needed to obtain such a level (the sets without washing were 1, 1HA, 4, and 4HA). The extraction of BPA-DAC was between 31 and 65% (medium 48%), and the acceptable level of 80% was not reached. The extraction of BPA-DMA and BPA-DGE by SPE was ineffective, possibly due to the sorption of these analytes to the analytical equipment. Hydrolysis could also be an issue. On the other hand, the duration of the extraction (pretreatment + SPE) was not more than 4 h, thereby much lower than the reported half-life of BPA-DGE in water (4.6 days (Lane et al. 2015)). The other reason for the low extraction of BPA-DMA and BPA-DGE is their high quantification limits by GC, mentioned in the previous paragraph. The application of the PSA sorbent did not disturb the extraction of BPAs, yet it was visually observed that it retained HAs (Fig. S4, Supplementary Information), thereby minimizing their presence inside the final extract. In the case of the analysis of WW samples, the use of PSA can lower the matrix effect in the GC analysis. In our previous work, the matrix effect in GC/MS was tested and was found to be connected mostly with the injection to GC (Caban et al. 2012), and any reduction in the injected extract composition was found to be beneficial for quantification by GC.

### Parameters of the validated SPE-GC/MS(SIM) analysis of the target bisphenols

The SPE-GC/MS(SIM) method was developed for the purpose of the study and was not a modification of any existing method. The parameters of the validated method are presented in Table 2, and are applicable for both SW and WW. The MQLs were between 1 and 5 ng/L, except for BPA-DAC, for which the MQL was 50 ng/L. The highest calibration level was 1000 ng/L for each of the BPs. For BPA-DMA and BPA-DGE, the methodological parameters were not presented because of their low extraction recovery from water samples by SPE; they were rejected from the group of analytes. In the blank samples, BPA was detected with a concentration < MQL. The within-day method recovery was acceptable, and the range of recovery values for all tested levels was 87–133%. The precision, presented as RSD, was below 13%.

Generally, the MQLs of the analysis of BPA by the LC/MS and GC/MS techniques are similar (Ros et al. 2015). Table 3 presents a comparison of the method developed here to those found in the literature for more than 7 analogues of BPs in one run. The recoveries in this work are generally higher. The MQLs are within the literature values (resulting from the sample volume used for the analysis).

**Table 3:** The comparison of the MQLs and recoveries from the analysis of bisphenol analogues determined in this work to others found in the literature

Number of bisphenols: targets	Method	Sample volume	MQL [ng/L]	Method recovery [%]	Reference
10: BPAF, BPE, BPF, BPA, BPC, BPS, BP-DAC, BPZ, BPBP, BPFL	SPE-GC/MS(SIM)	100 mL	1–5 (except 50 for BP-DAC)	87–133	This work
8: BPAF, BPAP, BPB, BPC, BPE, BPF, BPS, BPZ	SPE-GC/MS(SIM)	2 L	0.465–4.13	56–100	Česen et al. (2018)
14: BPA, BPF, BPS, BPAF, BPAP, BPP, BPB, BPZ, BPA-DGE (six related compounds)	SPE-LC/MS/MS	100 mL	1–100	61–117	Xue and Kannan (2019)
7: BPA, BPAF, BPB, BPE, BPF, BPS, BPZ	SPE-LC/MS/MS	500 mL	0.043–2.43	43–90	Sun et al. (2017)

### The concentrations of BPs found in wastewaters

In Table 4, the concentrations of BPA and BPS (ng/L), the only analytes found, are presented (rounded to a number with no decimal places). Some of them were above the upper validation level of 1000 ng/L. The substantial lower concentrations were found in the treated WWs compared with the raw WWs, which is in accordance with literature information regarding the relatively high performance of the active sludge process in the removal of bisphenols (Wang et al. 2019).

**Table 4:** The concentrations of BPA and BPS (ng/L) found in the tested samples of wastewater (WW) in comparison to the literature data

Compound	WWTP Gdansk-Wschód, Poland		WWTP Gniewino, Poland		WWTP Swarzewo, Poland		WWTP New York, USA, 2018 (Xue and Kannan 2019)		WWTP Xiamen, China, 2016 (Sun et al. 2017)	
	Raw WW	Treated WW	Raw WW	Treated WW	Raw WW	Treated WW	Raw WW	Treated WW	Raw WW	Treated WW
BPA	1465	42	1194	16	782	61	< MQL-8420	< MQL-3340	Median 1318	Median 177
BPS	1249	10	595	< MDL	1045	< MDL	< MQL-649	MQL-420	Median 48	Median 4

In the study performed in Ljubljana (Česen et al. 2018), from the eight tested BPs (without BPA measurement), the only one found in treated WW was BPS (40 ng/L), similar to our work. In the review from 2018, it was presented that BPA and BPS are the BPs with the highest concentrations worldwide, while the other ones (BPAF, BPAP, BPB, BPP, BPF, BPZ) are sporadic (Noszczyńska and Piotrowska-Seget 2018). BPA, BPS, and BPF were found with similar detection frequency in the WW sampled in China (Song et al. 2014). In our work, BPF was found to be below the MDL.

BPS is currently used as a safe replacement for BPA (Becerra and Odermatt 2012), but there are reports which present its negative impact on human health (Thoene et al. 2018; Qiu et al. 2019). BPS has an EDC character, compared with BPA (lower or higher depending on the study (Chen et al. 2016; Hąc-Wydro et al. 2019; Lee et al. 2019)). There are several origins of BPS in WWs. One of them is recycled paper used as toilet paper (Pivnenko et al. 2018). The application, occurrence, safety, and biodegradation of BPs are presented in the review by Noszczyńska and Piotrowska-Seget (Noszczyńska and Piotrowska-Seget 2018). The ecotoxicology of mixtures of BPs shows a significant endocrine threat (Owczarek et al. 2018). Thereby, the monitoring of BPA together with its analogues should be performed more often.

### The concentrations of BPs found in surface water

In Table 5, the concentrations of BPA and BPS are presented—similarly to the WW samples, only those two analytes were detected. The chromatographic peaks of BPA and BPS in blanks, standard samples, and various extracts are presented in Fig. S5 (Supplementary Information). The concentrations found were relatively high compared with the previously presented concentrations for WWs (Table 4), but lower than the literature data for SW, presented in Table 5. Special attention should be given to sample 2, as Kozacki Stream is partially supplied by water from the landfill (its location is presented in Fig. S2, Supplementary Information). In sample 3, the concentration of BPA was 3113 ng/L, thereby about twice as high as in raw WW from Gdansk-Wschód WWTP (Table 4). In the work performed in 2019 in the mentioned landfill, BPA was found in a concentration of between 856 and 2202 μg/L in the leachate from the old cell (currently not operated, with old barrier technology) (Wilk et al. 2019), about 8 times higher than the concentration in our study. The presence of the accelerated concentration of BPA in Kozacki Stream can suggest that the efforts performed by landfill operators (building of stream bypasses and barrier wells) have not totally eliminated the problem.

**Table 5:** The concentrations of BPA and BPS found in the four tested samples of surface water in comparison with the literature data (*NT*, not tested; *ND*, not detected)

Compound	Sample 1 (Oruński Stream)	Sample 2 (Kozacki Stream)	Sample 3 (outflow from Świętokrzyska I retention tank)	Sample 4 (outflow from Świętokrzyska II retention tank)	Leachate from landfill—Gdańsk, Poland (Wilk et al. 2019)	Leachate from landfill (Yamamoto et al. 2001)	Runoff and landfill leachate (Kalmykova et al. 2013)	Surface water (two lakes in China) (Yan et al. 2017)	Surface water and sea water samples from Japan, Korea, China, and India (Yamazaki et al. 2015)
BPA	170 ng/L	3113 ng/L	798 ng/L	207 ng/L	856–2202 μg/L (old cell of landfill)	1.3 to 17,200 μg/L with a median concentration of 269 μg/L	0.01–107 μg/L with a median concentration of 0.55 μg/L	28–560 ng/L	ND-1,950 ng/L
BPS	122 ng/L	> MDL ng/L	1584 ng/L	> MDL	NT	NT	NT	4–1600 ng/L	ND-7,200 ng/L

On the other hand, BPS was not detected but was present in sample 1 taken from the second stream which flows into the Świętokrzyski I retention tank. In this tank, both BPA and BPS were detected. From the Świętokrzyski I tank, the water flows into the Świętokrzyska II tank, where only BPA was detected. Sample 4 has a relatively low concentration of suspensions compared with samples 1–3 (judging by residues on the filters presented in Fig. S6, Supplementary Information), which means that the clarification of the water in this reservoir lowers the concentration of BPs. Bearing in mind the possibilities of BPs to be adsorbed in suspended matter, it is important to analyze whole water samples of these pollutants.

## Conclusions

This study shows the advantages and disadvantages of GC/MS as a tool for the quantification of mixtures of BPs. There are limits regarding the application of GC for BPA diglycidyl ether, BPA diacetate, and BPA dimethacrylate—compounds without the possibilities of trimethylsilylation, as they can decompose in the hot injector. The TMS derivatives of BPs give good mass spectra for qualitative and quantitative analysis by the SIM mode of mass spectra recording. The application of the PSA sorbent improves SPE—reducing the matrix component without a reduction in the extraction recovery of analytes. The validation of SPE-GC/MS(SIM) has proved its applicability for the determination of whole water concentrations of BPs in WW and SW. Despite the omnipresence of BPA, BPS was found in levels similar to BPA. This should raise attention since BPS has a similar EDC character to BPA, but is rather overlooked in risk assessment.

## Electronic supplementary material

ESM 1 (DOCX 30648 kb)
