# Supplementary material for: The quantification of bisphenols and their analogues in wastewaters and surface water by an improved solid-phase extraction gas chromatography/mass spectrometry method
Source: Environ Sci Pollut Res Int. 2020 May 16;27(23):28829–39. doi: 10.1007/s11356-020-09123-2 (PMC7375991; doi:10.1007/s11356-020-09123-2)
Supplement: Supplementary file 1 — (DOCX 30648 kb) [file 11356_2020_9123_MOESM1_ESM.docx]

Supplementary information to

**The quantification of bisphenols and their analogues in wastewaters and surface water by an improved solid-phase extraction-gas chromatography/mass spectrometry method**

Magda Caban*, Piotr Stepnowski

*Department of Environmental Analysis, Faculty of Chemistry, University of Gdansk, ul. Wita Stwosza 63, 80-308 Gdańsk, Poland*

***Corresponding author**: Phone (+48 58) 523 5208; E-mail address: magda.caban@ug.edu.pl

Section S1. Fragmentation of TMS derivatives of bisphenols

The fragmentation pattern of silyl derivatives of bisphenols follows the general schema for TMS derivatives, which were presented in **Fig. 1** and **Fig. 2**. The M**^+.^** molecular ion is observable with moderate intensity. The other pattern found is the breakdown of the band in the beta position to the aromatic ring. The M-15 signal came from the release of a methyl group. In the case of deuterated BPA (IS), M-18 was present, which suggests the release of a methyl group is another beta-fragmentation, rather than a fragmentation of the TMS group. The 73 m/z signal comes from the TMS group, and was the most intense or one of the most intense. This fact means that the TMS group is easily ionized and can be a reason for better TIC intensity of TMS-derivatives compared to underivatized bisphenols.

Table S1. Basic characteristic of the target bisphenol A, its analogues and internal standard

| No | Name [abbreviation] | Molar mass [g/mol]  Molecular formula  CAS | logP |
| --- | --- | --- | --- |
| 1 | Bisphenol E [BPE]  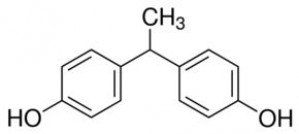 | 214.26  CH_3_CH(C_6_H_4_OH)_2_  2081-08-5 | 3.9 |
| 2 | Bisphenol C [BPC]  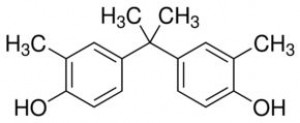 | 256.34  (CH_3_)_2_C[C_6_H_3_(CH_3_)OH]_2_  79-97-0 | 4.7 |
| 3 | Bisphenol A dimethacrylate  [BPA-DMC]  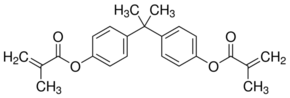 | 364.43  [H_2_C=C(CH_3_)CO_2_C_6_H_4_]_2_C(CH_3_)_2_  3253-39-2 | 6.2 |
| 4 | Bisphenol BP [BPBP]  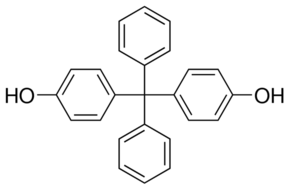 | 352.43  C_25_H_20_O_2_  1844-01-5 | 5.6 |
| 5 | Bisphenol F [BPF]  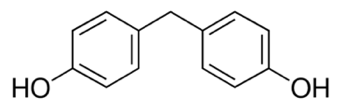 | 200.23  CH_2_(C_6_H_4_OH)_2_  620-92-8 | 2.9 |
| 6 | Bisphenol A diglycidyl ether [BPA-DGE]  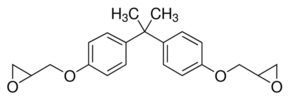 | 340.41  C_21_H_24_O_4_  1675-54-3 | 4.0 |
| 7 | Bisphenol A diacetate, 98% [BPA-DAC]  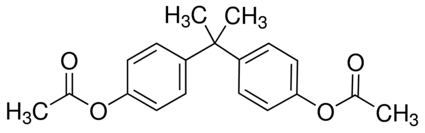 | 312.36  (CH_3_CO_2_C_6_H_4_)_2_C(CH_3_)_2_  10192-62-8 | 4.2 |
| 8 | Bisphenol Z [BPZ]  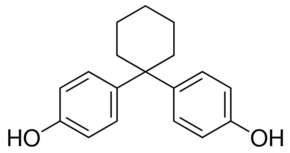 | 268.35  C_6_H_10_(C_6_H_4_OH)_2_  843-55-0 | 5.4 |
| 9 | Bisphenol FL [BPFL]  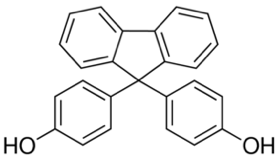 | 350.41  C_25_H_18_O_2_  3236-71-3 | 6.0 |
| 10 | Bisphenol AF [BPAF]  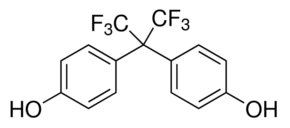 | 336.23  (CF_3_)_2_C(C_6_H_4_OH)_2_  1478-61-1 | 4.5 |
| 11 | Bisphenol S [BPS]  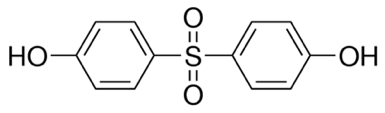 | 250.27  O_2_S(C_6_H_4_OH)_2_  80-09-1 | 1.9 |
| 12 | Bisphenol A [BPA]  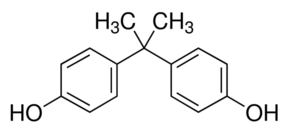 | 228.29  (CH_3_)_2_C(C_6_H_4_OH)_2_  80-05-7 | 3.32  120 mg/L |
| 13 | Internal standard: Bisphenol A-D16 [BPA-D16]  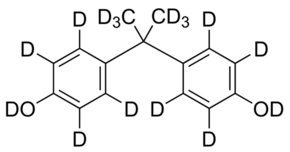 | 244.38  (CD_3_)_2_C(C_6_D_4_OD)_2_  96210-87-6 | - |

(log P – pubchem.org database, mostly calculated)

Table S2. The GC/MS(SIM) instrumental calibration parameters

| Analyte  Qualitative/  Reference m/z | Retention time  [min] | Instrumental / method correlation coefficient (R^2^) | Precision (RSD)  Instrumental / method [%] | Instrumental trueness  (C_found/_C_nominal_ x100%) [%] | Instrumental quantification limit (IQL)  [ng/mL] | Instrumental detection limit (IDL)  [ng/mL] |
| --- | --- | --- | --- | --- | --- | --- |
| BPAF  411/480, 465 | 12.55 | 0.9999 | 1.1-6.2 | 102-106 | 19.5 | 6.5 |
| BPF  344/157 | 13.90 | 0.9999 | 1.1-4.7 | 99-102 | 19.5 | 6.5 |
| BPE  343/358 | 14.81 | 0.9999 | 1.0-4.7 | 95-110 | 19.5 | 6.5 |
| BPA  357/372 | 14.51 | 1.0000 | 1.2-3.5 | 99-104 | 19.5 | 6.5 |
| BPC  385/400 | 15.20 | 0.9998 | 1.5-6.1 | 100-105 | 19.5 | 6.5 |
| BPA-DAC  213/228, 270, 312 | 15.88 | 0.9985 | 1.3-8.1 | 88-101 | 78.1 | 26.0 |
| BPZ  412/369, 343 | 17.76 | 0.9996 | 1.3-6.6 | 93-102 | 19.5 | 6.5 |
| BPS  394/379, 182 | 18.40 | 0.9990 | 1.6-11.4 | 99-104 | 39.1 | 13.0 |
| BPA-DMA  349/364 | 18.97 | *0.9891* | 0.9-7.8 | *98-115* | *156.2* | *52.1* |
| BPA-DGE  325/340 | 20.25 | *0.9885* | 5.1-7.7 | *90-109* | *1250.0* | *416.7* |
| BPBP  419/331, 496 | 22.54 | 0.9996 | 1.2-5.0 | 100-103 | 78.1 | 26.0 |
| BPFL  494/329 | 24.45 | 0.9995 | 0.6-5.4 | 99-107 | 39.1 | 13.0 |


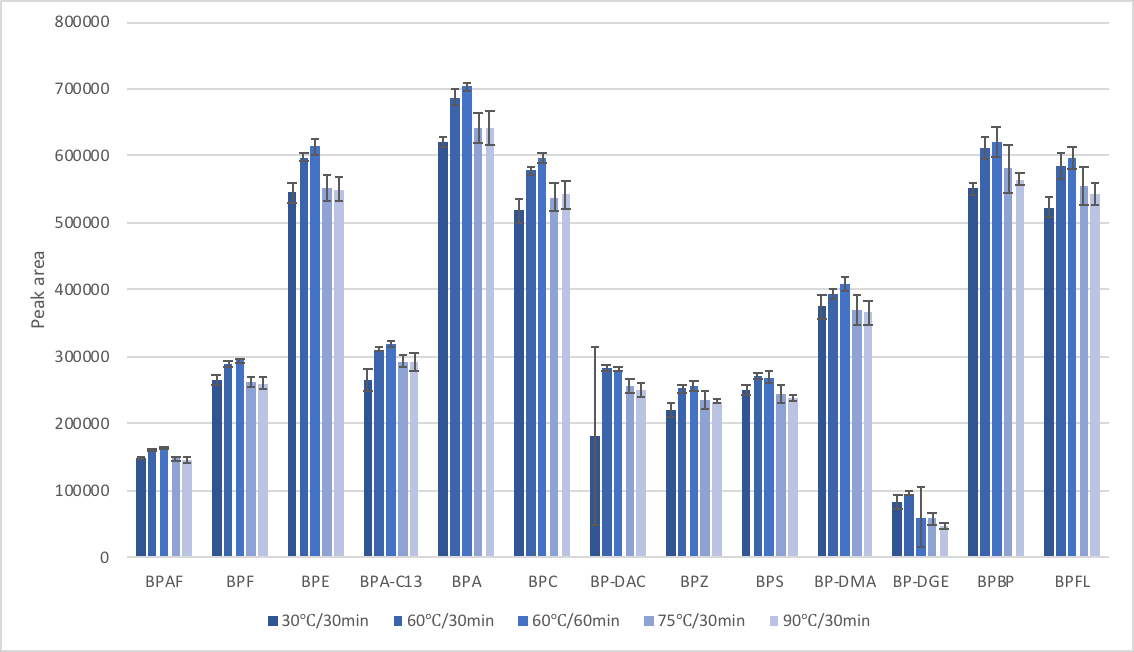


Figure S1. The peaks area (GC/MS(SIM)) of target bisphenols obtained in different temperature and time of silylation by BSTFA reagent


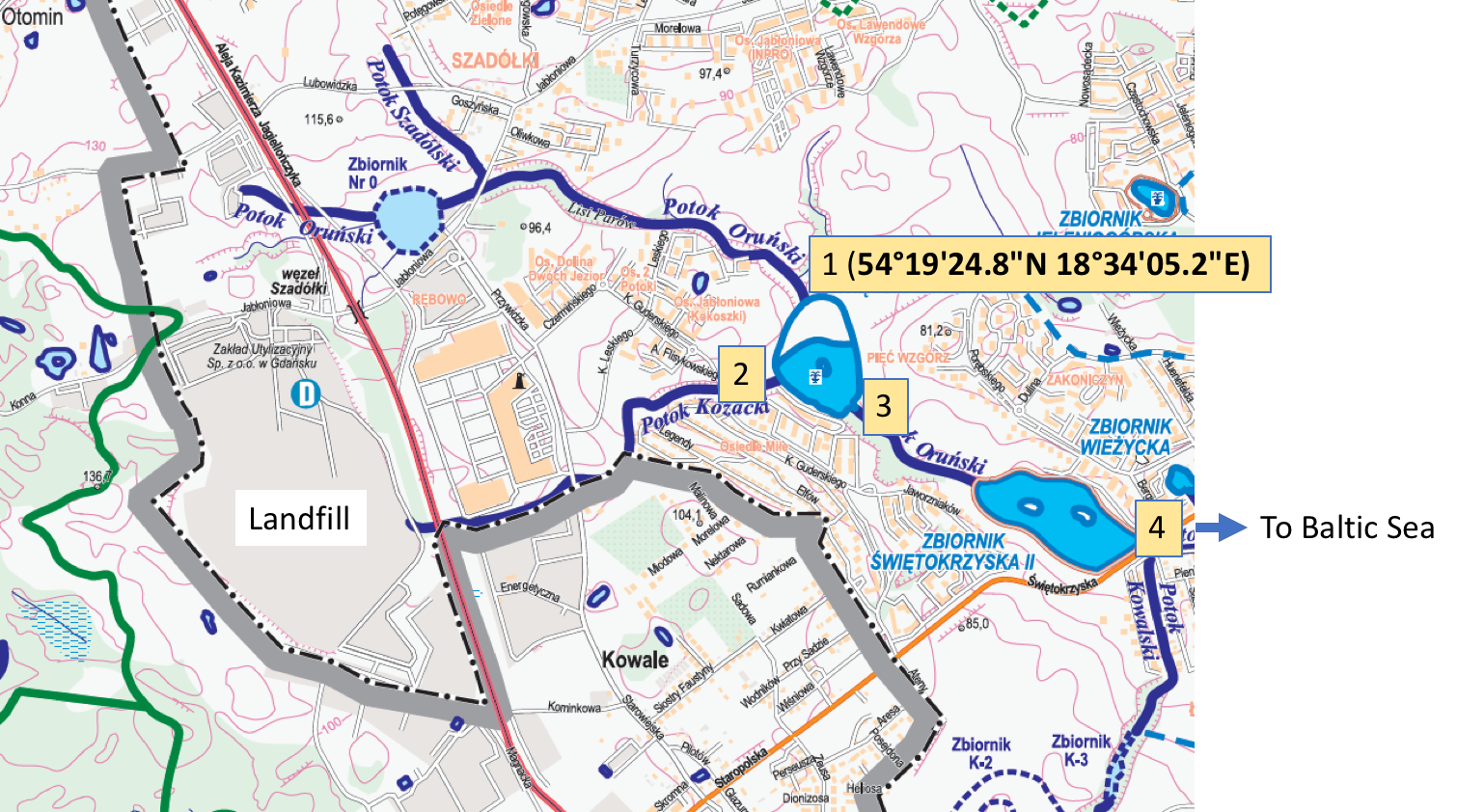


Figure S2. The map with four sampling points (6.01.2020) of surface water in Gdańsk (Poland)

(www.gis.gdansk.pl)


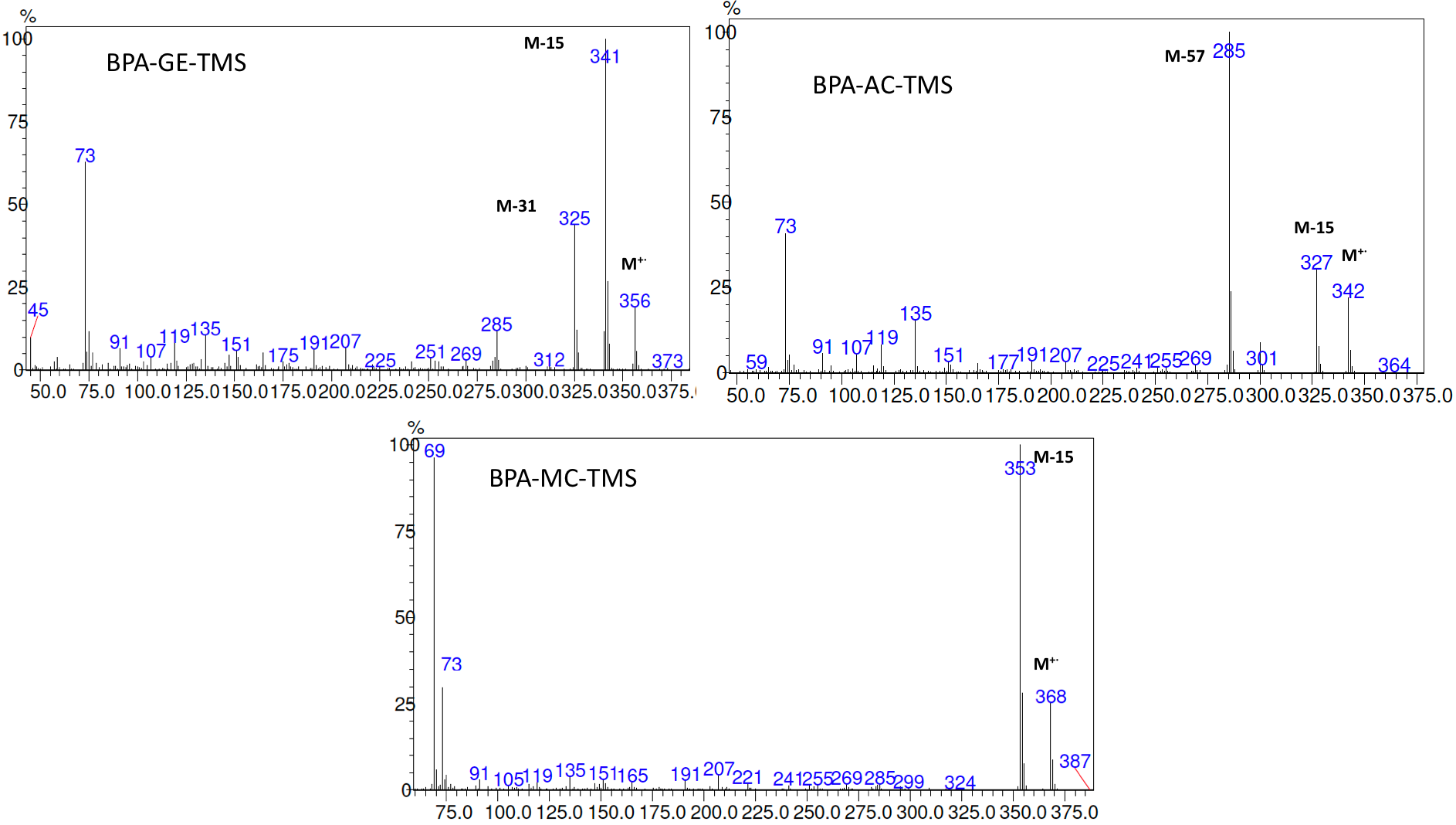


Figure S3. The mass spectra of mono-TMS derivative of the BPA-methacrylate-mono-*O*-TMS (BPA-MC-TMS), BPA-glycidyl ether-mono-*O*-TMS, and BPA-acetate-mono-*O*-TMS


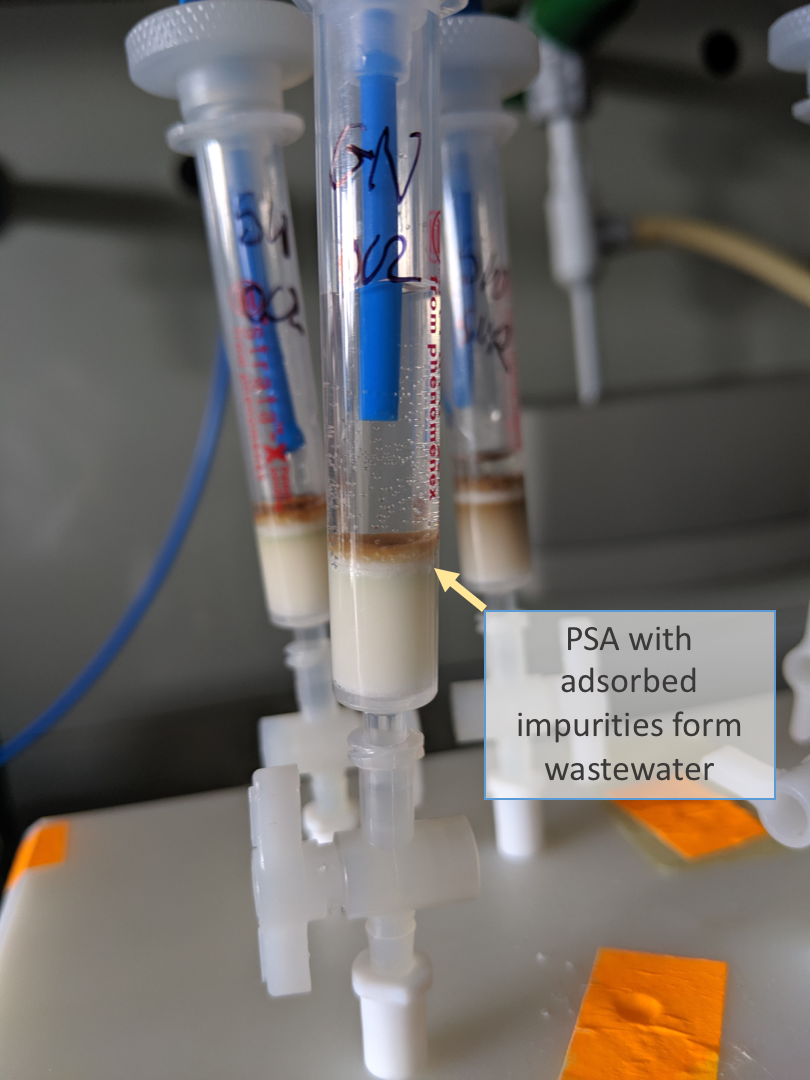


Figure S4. Photography of Strata-X column with PSA sorbent on the top of original sorbent with visible adsorbed impurities from wastewater sample


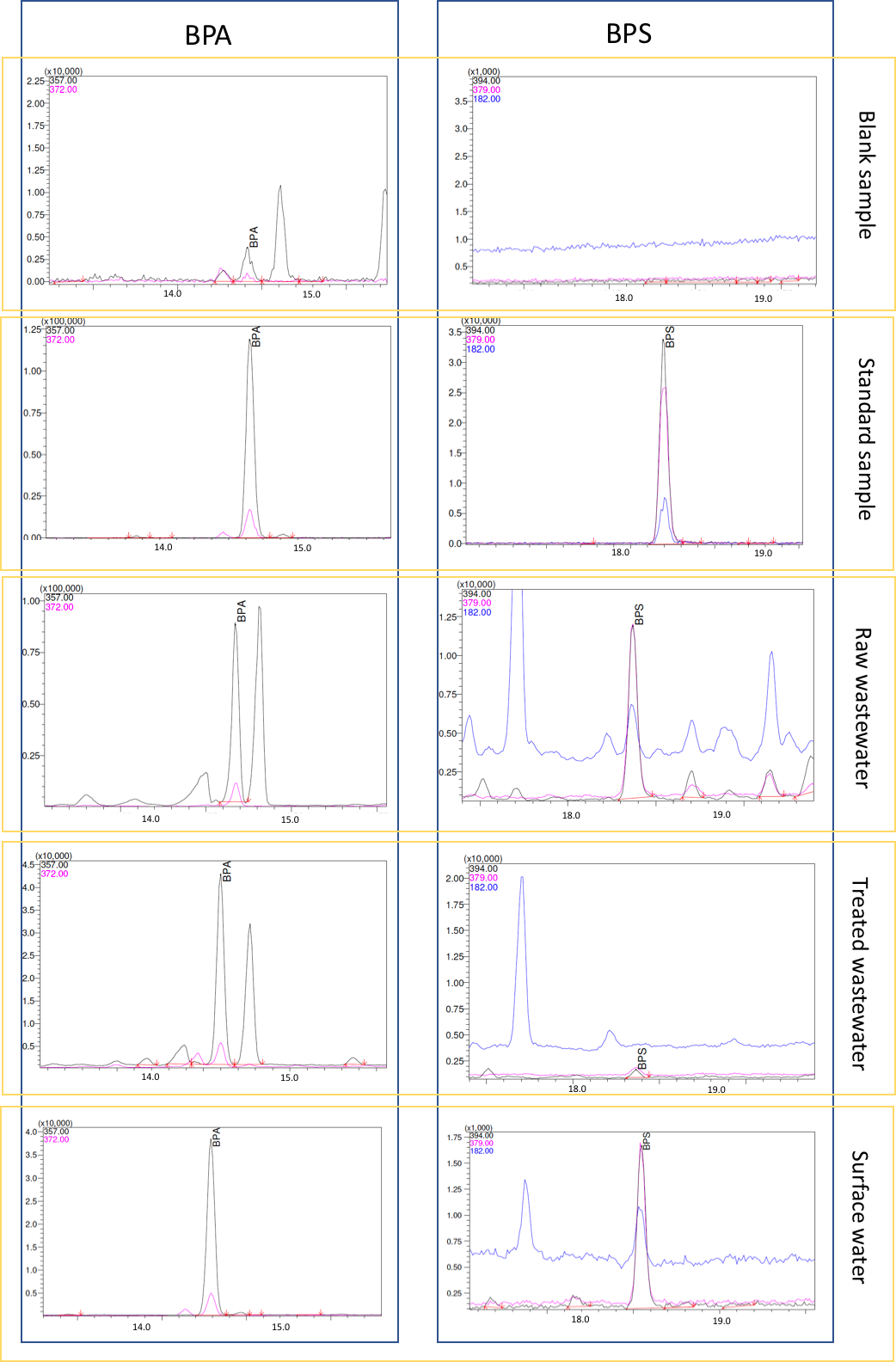


Figure S5. The fragments of SIM chromatograms presented detection of bisphenol A (BPA) and bisphenol S (BPS) in blank sample, standard sample, and extracts of raw and treated wastewater (WWTP Gniewino) and surface water (Oruński Stream)


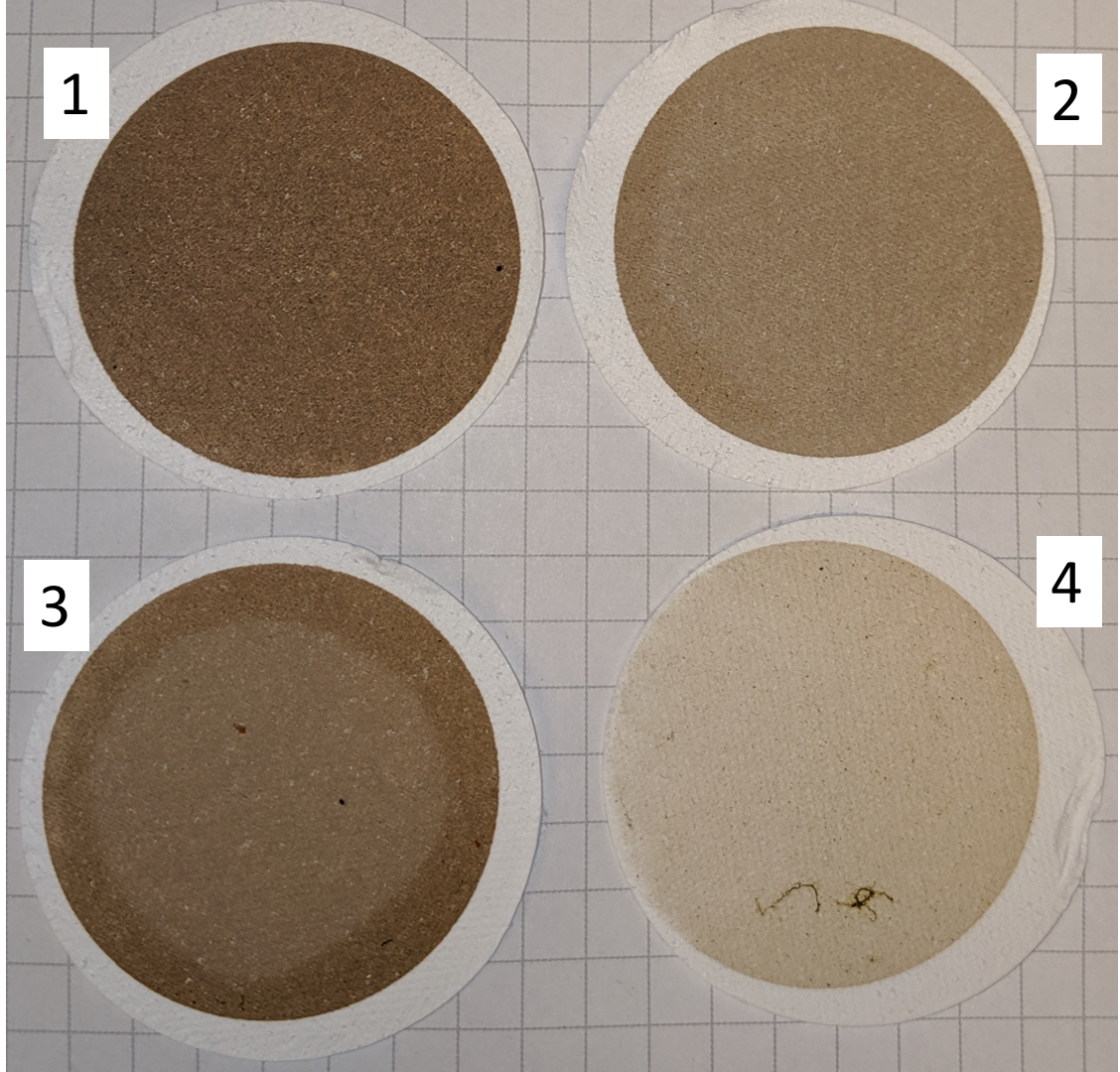


Figure S6. The glass fiber filters after filtration of surface water 1-4 sampled for this study
